# Adaptability of lung and liver metastatic breast cancer cells to glucose

**DOI:** 10.1186/s12935-025-04006-3

**Published:** 2025-10-24

**Authors:** Marjorie Anne Layosa, Madeline P. Sheeley, Alekya Raghavan, Chaylen Andolino, Michael K. Wendt, Stephen D. Hursting, Dorothy Teegarden

**Affiliations:** 1 Department of Nutrition Science, College of Health and Human Sciences, Purdue University, West Lafayette, United States; 2 Purdue University Institute for Cancer Research, Purdue University, West Lafayette, United States; 3 Department of Biological Sciences, College of Science, Purdue University, West Lafayette, United States; 4 Department of Internal Medicine, Carver College of Medicine, University of Iowa, Iowa, United States; 5 Holden Comprehensive Cancer Center, University of Iowa, Iowa, United States; 6 Department of Nutrition, Gillings School of Public Health, University of North Carolina at Chapel Hill, Chapel Hill, United States; 7 Lineberger Comprehensive Cancer Center, University of North Carolina at Chapel Hill, Chapel Hill, United States

**Keywords:** Breast cancer, Metastasis, Metabolic adaptation, Glucose

## Abstract

**Background:**

Breast cancer is the most common cancer among women, and metastasis is the leading cause of mortality. It is still unknown how breast cancer cells metabolically adapt to successfully metastasize to different organs to survive adverse conditions, including varying nutrient availability. The purpose of this study is to elucidate the metabolic characteristics and glucose adaptation mechanisms of breast cancer cells that preferentially metastasize to the lungs or the liver.

**Methods:**

Using a Wnt-driven breast cancer model with preferential metastasis to lung (metM-Wnt^Lung^) or liver (metM-Wnt^Liver^), we measured ^14^C-glucose uptake, ^13^C_6_-glucose metabolic flux, metabolic enzyme levels, and cell viability under normal (5 mM), high (25 mM), and low (1 or 0 mM) glucose conditions.

**Results:**

Under normal glucose conditions, metM-Wnt^Lung^ cells were more glycolytic, exhibiting greater flux of ^13^C_6_-glucose-derived carbons into glycolytic intermediates, such as pyruvate and lactate. In contrast, metM-Wnt^Liver^ cells favored oxidative phosphorylation, with higher levels of ^13^C_6_-glucose-derived carbons in tricarboxylic acid (TCA) cycle metabolites such as oxaloacetate indicative of higher pyruvate carboxylase (PC) activity. Exposure to high glucose reduced metM-Wnt^Liver^ cell viability, with no effect on metM-Wnt^Lung^ cells, suggesting better adaptability of metM-Wnt^Lung^ cells to glucose excess. This was accompanied by increased PC activity and oxidative phosphorylation in metM-Wnt^Lung^ cells, whereas metM-Wnt^Liver^ cells shifted to a more glycolytic phenotype. Under glucose deprivation, metM-Wnt^Lung^ cells were more viable than metM-Wnt^Liver^ cells, suggesting that metM-Wnt^Lung^ cells have better adaptability to glucose deprivation. Inhibiting phosphoenolpyruvate carboxykinase, a key enzyme in gluconeogenesis, reduced metM-Wnt^Lung^ cell viability compared to metM-Wnt^Liver^ cells. Similarly, inhibiting catabolism of glutamine, a gluconeogenic substrate, decreased metM-Wnt^Lung^ cell viability compared to metM-Wnt^Liver^ cells, indicating that metM-Wnt^Lung^ cells rely on more on gluconeogenesis and glutamine metabolism under glucose deprivation.

**Conclusion:**

Our findings reveal that metM-Wnt^Lung^ cells exhibit greater metabolic flexibility to glucose than metM-Wnt^Liver^ cells by shifting from glycolysis to oxidative phosphorylation under high glucose conditions while utilizing gluconeogenesis and glutamine under glucose deprivation conditions.

## Introduction

Breast cancer is the most common cancer among women, accounting for 31% of all female cancers, and metastases are responsible for the majority of deaths from the disease [1, 2]. Reprogramming of energy metabolism, a cancer hallmark, includes cellular adaptations necessary to sustain cellular proliferation and survival [3–5]. Metabolic reprogramming is critical to establish successful metastases when cancer cells are exposed to environments with varied nutrient availability, oxidative stresses, and other barriers to colonization at different sites [4, 6–8]. Thus, it is important to investigate how cells metabolically reprogram to identify strategies capable of preventing or treating metastasis, thereby improving survival and quality of life of breast cancer patients.

Glucose is a primary nutrient source that provides not only ATP but also carbon backbones for an array of biomolecules among which are fatty acids and cholesterol, pentose and hexose sugar derivatives, glycerol, nucleotides, and nonessential amino acids [3]. A markedly increased uptake and utilization of glucose regardless of oxygen availability (aerobic glycolysis), termed the ‘Warburg effect’, is well characterized in multiple types of human tumors [3, 5, 9–11]. Increased glycolysis confers an advantage to tumor cells by generating glycolytic intermediates that can be diverted for cellular requirements via the pentose phosphate pathway, hexosamine biosynthesis, phospholipids biosynthesis, and one-carbon cycle to generate diverse biosynthetic precursors [3, 9]. However, mitochondrial metabolism through the tricarboxylic acid (TCA) cycle, a central hub of energy metabolism integrating glucose, glutamine, and fatty acid metabolism, is indispensable to some cancer types such as melanoma, KRAS-driven lung adenocarcinoma, brain tumor, and circulating tumor cells. Mitochondrial metabolism generates metabolic precursors that funnel into nucleotide, lipid, amino acid, and heme synthesis, and ATP production [3, 11]. Although glycolysis and mitochondrial metabolism are often negatively correlated in normal tissues, these processes may be concurrent in cancer cells, enabling metabolic plasticity to support survival in diverse environments [2, 9, 12, 13]. While glucose provides the main source of pyruvate entering the TCA cycle in normal cells, cancer cells often shunt glucose away from the TCA cycle for catabolism, and thus depend on other nutrients such as glutamine and fatty acids to replenish TCA cycle intermediates [14]. Such findings indicate that different cancer types preferentially utilize diverse alternative pathways and that the availability of glucose may influence the overall metabolism and adaptation of cancer cells to successfully metastasize [15].

Evidence supports that metabolic reprogramming may drive organ-specific metastasis [2, 6, 7, 16–19]. Metastasis is facilitated by multiple factors, including the ability of cancer cells to adapt to the changing environments encountered during metastasis [12, 15]. The lungs and liver, two common sites of metastatic breast cancer, represent distinct microenvironments to which cancer cells must adapt in order to successfully metastasize. For instance, the lung is highly oxygenated with pyruvate-enriched interstitial fluid while the liver is a metabolic center with heterogenous oxygen levels and unique roles in detoxification [7, 20]. Indeed, primary tumor cells and metastatic cells found in different sites display distinct metabolic profiles, suggesting that metabolic reprogramming is a feature of successful and preferential metastasis to different organs [16, 19, 21]. For example, MDA-MB-231-derived lung and bone-homing cells are more glycolytic and have lower ATP demand compared to the parent cell line, suggesting that stable metabolic alterations can occur as primary breast cancer cells metastasize [19]. Further, 4T1-derived liver metastatic breast cancer cells exhibit higher glycolytic activity and pyruvate-to-lactate conversion compared to subsequent bone and lung metastases [16]. These findings indicate that metastasis to different sites may require distinct metabolic characteristics and compatibility with the immediate microenvironment to support organ-specific metastasis.

Despite clear evidence for metabolic reprogramming of tumor cells to permit metastasis, investigation of metabolic differences of syngeneic cells for preferential metastasis to different sites remains largely unexplored. Further, adaptations in the context of organ-specific metastasis under varying glucose concentrations has yet to be studied. Understanding how breast cancer cells with preferential metastasis to different sites adapt their glucose metabolism under varying environmental stresses may contribute to identifying and targeting potential metabolic vulnerabilities to reduce, if not prevent, breast cancer progression. We hypothesize that glucose metabolism and adaptation are metabolically unique between lung- and liver-specific metastatic breast cancer cells to successfully survive stressful environmental conditions.

## Methodology

### Chemicals and antibodies

30% (w/w) hydrogen peroxide (H_2_O_2_) in water, oxaloacetic acid, 2-deoxy-D-glucose (2DG), glutaminase inhibitor (GLS-968), and dimethyl sulfoxide (DMSO) were purchased from Sigma-Aldrich (St. Louis, MO). The cytosolic phosphoenolpyruvate carboxykinase inhibitor (PCKi) was purchased from Axon Medchem (Reston, VA). Antibodies for pyruvate dehydrogenase (PDH), phospho-PDH, and actin were purchased from Cell Signaling Technology Inc. (Danvers, MA), and pyruvate carboxylase (PCB) was purchased from Abcam (Waltham, MA).

### Cell line model

The parental M-Wnt cell line was derived from the spontaneous primary tumor of a mouse mammary tumor virus Wnt-1 transgenic mouse and is non-metastatic [22]. Serial in vivo passaging of labeled M-Wnt cells for 5 generations in severe-combined immunodeficient mice resulted in two independent metastatic cell lines, metM-Wnt^Lung^ cells and metM-Wnt^Liver^ cells, that colonize the lung and liver, respectively [23]. In brief, M-Wnt cells were transduced with a GFP-Luciferase dual labeling lentiviral construct (Systems Bioscience), and 5 × 10^5^cells in 50 µl were injected into the fourth mammary fat pad of a severe combined immunodeficient (SCID) mice. The resulting mammary fat pad tumor was excised, and a tumor brei cell suspension was prepared as previously described [22], which was then injected into the 4th mammary fat pad of SCID mice (serial passage 1; *n* = 3/passage). This was repeated 4 additional times until lung and liver metastases were detected in atleast one of the mice, as occurred in the 4th passage. Following transplantation of the 5th passage, a survival surgery was conducted to remove the primary tumor, confirmed by in vivo imaging system (IVIS) imaging. Mice were monitored for luciferase-positive lung or liver metastases and once detected, tumors were harvested, cells were sorted by flow cytometry according to GFP expression, and the metM-Wnt^Lung^ and metM-Wnt^Liver^ cell lines were established and characterized [23].

### Cell culture

The cell series were maintained in modified Dulbecco’s Modified Eagle Medium (DMEM) containing 5 mM glucose, 2 mM glutamine, 10 mM HEPES, 3.7 g/L sodium bicarbonate, without sodium pyruvate, supplemented with 10% (v/v) fetal bovine serum (FBS) and 1% (v/v) penicillin–streptomycin antibiotic mix (Gibco, Billings, MT), and incubated at 37 °C with a humidified 5% CO_2_ atmosphere. The cells were maintained under specified levels of glucose concentrations for multiple passages, and thus represent stable traits, not acute changes.

### Gene expression

Cells (6.6 × 10^3^) were treated with or without the indicated treatments for 48 h. After the incubation period, RNA was harvested using TRI Reagent (Molecular Research Center, Cincinnati, OH) according to the manufacturer’s protocol. mRNA was reverse transcribed using the MMLV reverse transcriptase (Promega, Madison, WI) followed by amplification using LightCycler 480 SYBR Green I Master Mix (Roche, Indianapolis, IN) [24, 25]. Relative mRNA levels were calculated by comparative C_T_ method, as detailed previously [26]. The list of primer sequences for qRT-PCR are listed in Table 1.

**Table 1:** Primer sequences

Gene (mouse)	Sequence
18 S	Forward: 5’ ATC CCT GAG AAG TTC CAG CA 3’
	Reverse: 5’ CCT CTT GGT GAG GTC GAT GT 3’
Hexokinase (*Hk*)	Forward: 5’ TGA TCG CCT GCT TAT TCA CGG 3’
	Reverse: 5’ AAC CGC CTA GAA ATC TCC AGA 3’
Pyruvate Carboxylase (*Pc*)	Forward: 5’ TCA CCA GTG ACT CTG TCA AAC 3’
	Reverse: 3’ GAC CAG GTC CAC ATC TGT AAT 3’
Cytosolic Malic Enzyme 1 (*Me1*)	Forward: 5’ GTC GTG CAT CTC TCA CAG AAG 3’
	Reverse: 5’ TGA GGG CAG TTG GTT TTA TCTT 3’
Pyruvate Dehydrogenase Kinase 1 (*Pdk1*)	Forward: 5’ GGA CTT CGG GTC AGT GAA TGC 3’
	Reverse: 5’ TCC TGA GAA GAT TGT CGG GGA 3’

### Protein expression

Cells (3.3 × 10^5^) were treated with or without the indicated treatments for 48 h. After the incubation period, cells were washed with cold phosphate-buffered solution (PBS) then lysed and harvested using radioimmunoprecipitation assay (RIPA) buffer (Cell Signaling, Danvers, MA) containing 1% phenylmethanesulfonyl fluoride protease inhibitor (Cell Signaling, Danvers, MA) and phosphatase inhibitor cocktail 2 (Sigma-Aldrich, St. Louis, MO). Protein concentration of cell lysates was determined using Pierce bicinchoninic acid assay (BCA) protein assay kit (ThermoFisher, Waltham, MA). Equal amounts of protein (25 µg) were separated on polyacrylamide gels (7.5% or 10%) (Bio-Rad, Hercules, CA) and transferred to 0.2 μm nitrocellulose membranes (Bio-Rad, Hercules CA). Bands were visualized using SuperSignal West Pico PLUS Chemiluminescent Substrate reagent (ThermoScientific, Waltham, MA) and then imaged using x-ray film developed on an automatic x-ray film processor. Band intensities were quantified using ImageJ software.

### ^14^C-Glucose uptake

Cells (1.05 × 10^5^) were treated with or without the indicated treatments for 48 h and after the incubation period, medium was changed to media containing D-[^14^C(U)]-Glucose (0.25 µCi/mL) (PerkinElmer, Waltham, MA) and cells were incubated at 37 °C for 10 min. Cells were washed twice and harvested with cold PBS. The cell lysate was assessed using a Tri-Carb 5110TR 110 V Liquid Scintillation Counter (PerkinElmer, Waltham, MA) to detect incorporated ^14^C-label. A duplicate cell culture dish was incubated with 0.1 N NaOH overnight and protein quantified using the BCA protein kit. Radioactivity is expressed as nmol/µg protein.

### ^13^C-Glucose metabolic flux analysis

Cells (1.05 × 10^5^) were treated with or without the indicated treatments for 48 h. After treatments, media was replaced with fresh media containing [U-^13^C]glucose for 2 h at 37 °C. Cell harvest and sample derivatization were conducted as previously reported [27]. Briefly, dishes were rapidly washed with cold 1X PBS, scraped and collected using 70 °C 70% ethanol in water (v/v). Cells were vortexed, heated at 95 °C for 5 mins, and chilled on ice for 5 mins. Supernatants were collected after centrifugation at 14,000xg for 5 mins and dried under a stream of nitrogen gas. Dried samples were derivatized with 2% methoxylamine hydrochloride in pyridine solution (wt/vol) and N-tert-butyldimethylsilyl-N-methyltrifluoroacetamide with 1% (wt/wt) tert-butyldimethylchlorosilane. Samples were analyzed with gas chromatography mass spectrometry (Thermo TSQ 8000 triple quadrupole mass spectrometer coupled with a Thermo Trace 1310 gas chromatography). ^13^C metabolite level was calculated by dividing the total peak area of the labeled metabolite of interest to total peak area of all its isotopologues without normalization to an internal standard. Data is expressed as % of total pool following IsoCor correction [28].

### Viability

Viability was assessed using the 3-[4,5- dimethylthiazol-2-yl]−2,5-diphenyl tetrazolium bromide (MTT) assay (Sigma-Aldrich, St. Louis, MO) in serum-free media per the manufacturer’s instructions. Viability is expressed as relative absorbance.

### Statistical analysis

Results are expressed as mean values ± standard error of the mean (SEM) and represent at least three replicates. Data were analyzed by two-tailed distribution Student’s t-test and visualized using GraphPad Prism 10.0.0. P-value < 0.05 were considered statistically significant.

## Results

### Differential glycolytic metabolism in normal glucose concentrations

**Fig. 1:**
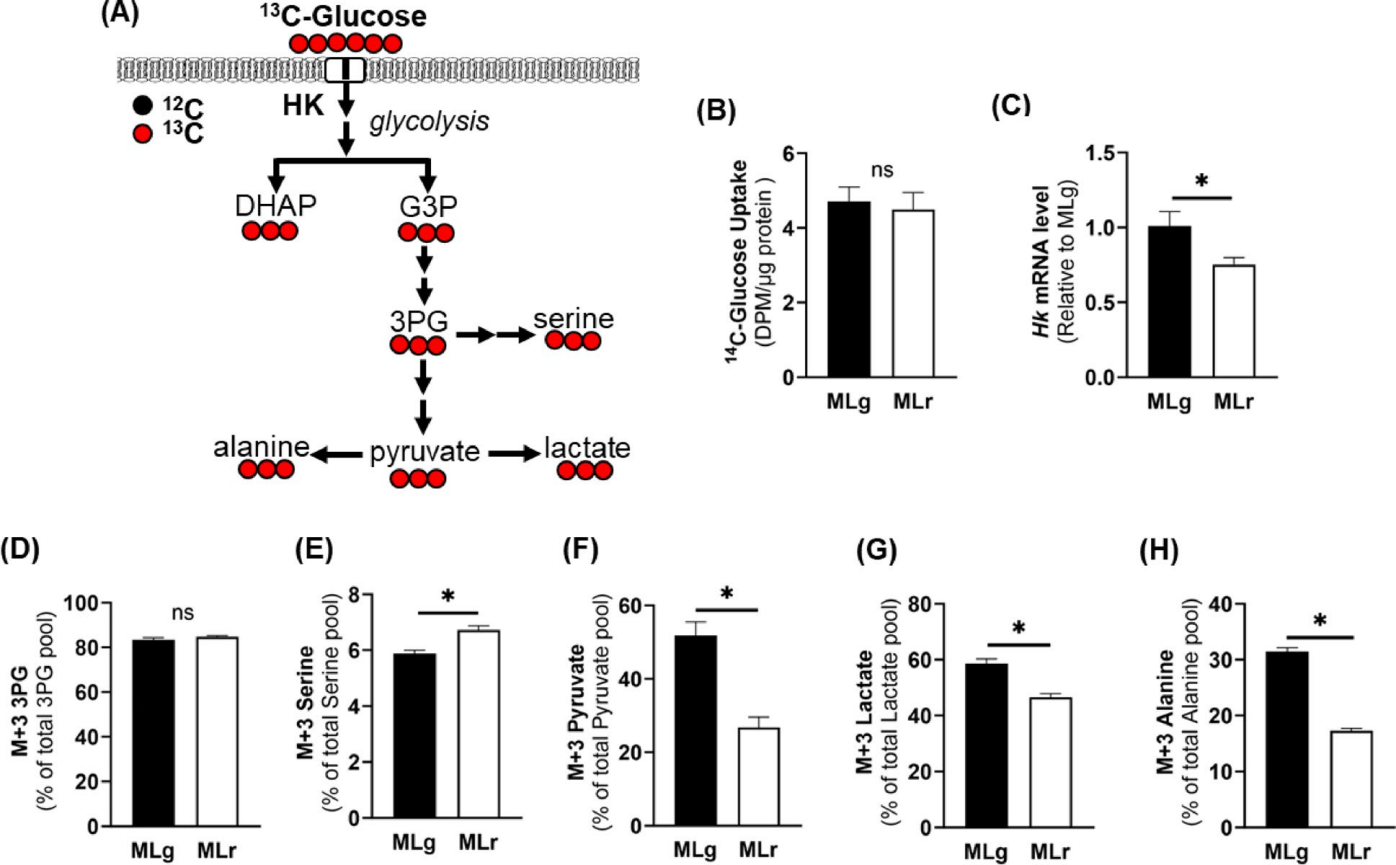
Glucose metabolism through glycolysis. (**A**) Schematic diagram of ^13^C_6_-glucose flux into glycolytic intermediates. (**B**) ^14^C-glucose uptake normalized to protein. (**C**) mRNA abundance of hexokinase normalized to 18 S level. ^13^C_6_-glucose incorporation into (**D**) M + 3-labeled phosphoglycerate, (**E**) M + 3-labeled serine, (**F**) M + 3-labeled pyruvate, (**G**) M + 3-labeled lactate, and (**H**) M + 3-labeled alanine. Data are presented as mean ± SEM (*n* = 4). MLg = metM-Wnt^Lung^; MLr = metM-Wnt^Liver^. * *p* < 0.05; ns – not significant

To evaluate differences in glucose metabolism between mammary tumor cells metastasizing to different organs, we utilized two previously characterized Wnt-driven breast cancer cell lines that preferentially metastasize either to the lungs or liver [23]. ^14^C-glucose uptake was similar in both metastatic cell lines (Fig. 1B). We also observed no difference in glucose uptake between the metastatic cell lines after a shorter incubation period (30 s) (data not shown). However, mRNA abundance of hexokinase (*Hk*), a rate-limiting enzyme in glycolysis, was 26% higher in metM-Wnt^Lung^ compared to metM-Wnt^Liver^ cells (*p* = 0.046) (Fig. 1 C). Furthermore, metabolic tracing of universally labeled ^13^C_6_-glucose revealed that although M + 3-labeled 3-phosphoglycerate (3PG) was similar between cell lines (Fig. 1D), M + 3-labeled serine was 20% higher in metM-Wnt^Liver^ compared to metM-Wnt^Lung^ cells (Fig. 1E), whereas M + 3-labeled pyruvate was 2-fold higher in the metM-Wnt^Lung^ compared to the metM-Wnt^Liver^ cells (Fig. 1 F). Consistent with these results, enrichment of M + 3-pyruvate-derived metabolites, lactate and alanine, were significantly higher in metM-Wnt^Lung^ cells (Figs. 1G–H). Overall, results indicate that metM-Wnt^Lung^ cells display a more glycolytic phenotype compared to the metM-Wnt^Liver^ cells.

**Fig. 2:**
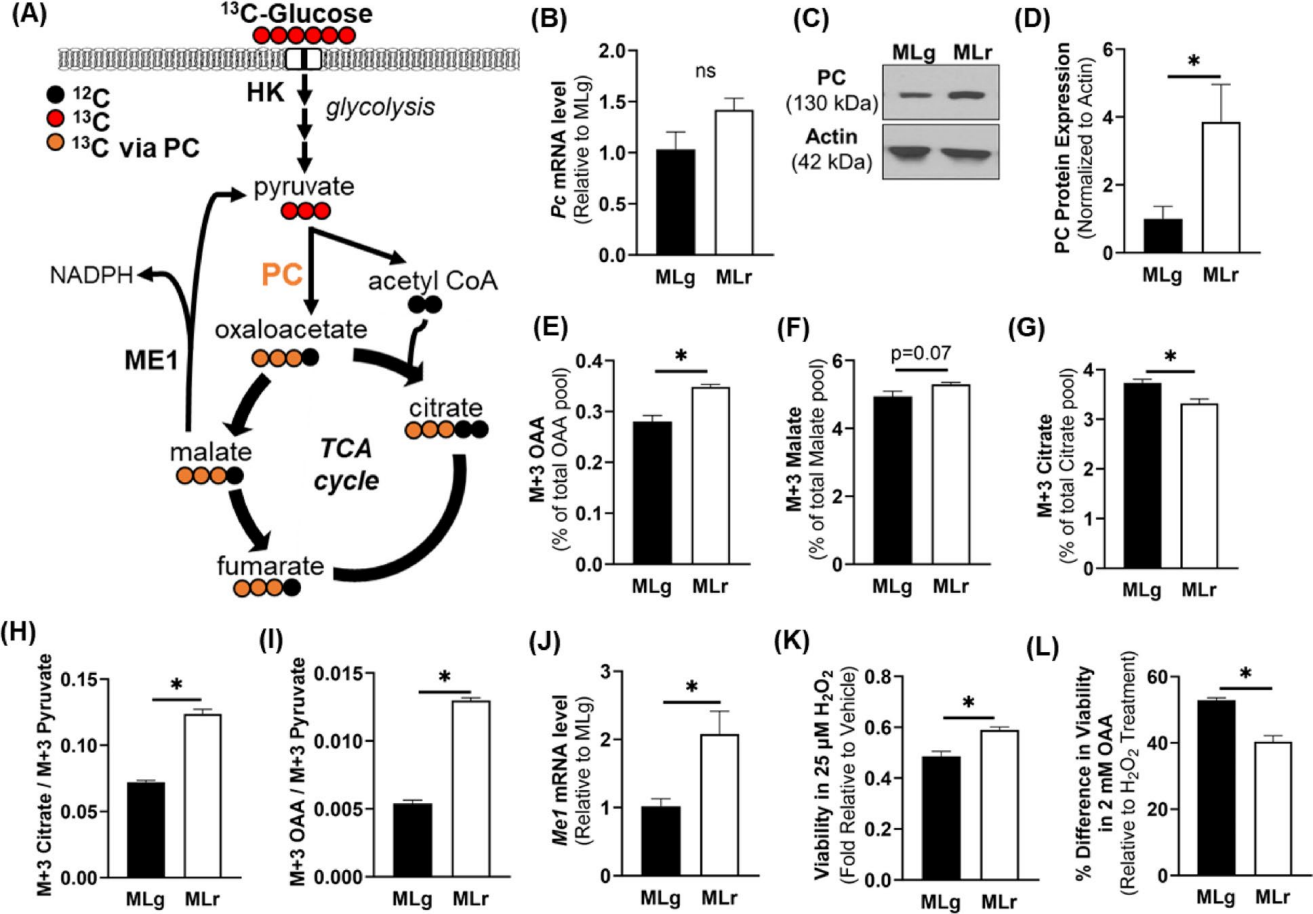
Glucose metabolism via pyruvate carboxylase into the tricarboxylic acid cycle. (**A**) Schematic diagram of ^13^C_6_-glucose flux into tricarboxylic acid cycle intermediates. (**B**) mRNA abundance of pyruvate carboxylase normalized to 18 S level. (**C**) Representative Western blot and (**D**) quantification of pyruvate carboxylase protein levels. All band intensities of target protein were normalized to actin.^13^C_6_-glucose incorporation into (**E**) M + 3-labeled oxaloacetate, (**F**) M + 3-labeled malate, and (**G**) M + 3-labeled citrate indicative of pyruvate carboxylase activity. (**H**) M + 3 citrate/M + 3 pyruvate and (**I**) M + 3 OAA/M + 3 pyruvate indicating pyruvate flux into tricarboxylic acid cycle through pyruvate carboxylase. (**J**) mRNA abundance of cytosolic malic enzyme 1 normalized to 18 S level. (**K**) Viability of the cell lines after treatment of 25 µM H_2_O_2_ for 24 h and (**L**) % change in viability after concurrent treatment of 2 mM OAA for 24 h. Data are presented as mean ± SEM (*n* = 4 for PCR, western blot and isotope tracing; *n* = 5 for viability assays). MLg = metM-Wnt^Lung^; MLr = metM-Wnt^Liver^. * *p* < 0.05; ns – not significant

We also compared the metabolism of pyruvate, the end-product of glycolysis, into the TCA cycle across cell lines. Although the mRNA abundance of pyruvate carboxylase (*Pc*), which converts pyruvate to oxaloacetate (OAA), was not significantly different (Fig. 2B), the PC protein level was approximately 3-fold higher in the metM-Wnt^Liver^ compared to metM-Wnt^Lung^ cells (*p* = 0.049) (Figs. 2C–D). Consistent with these results, enrichment of M + 3-labeled OAA, a product from pyruvate through PC conversion (Fig. 2A), was significantly higher in the metM-Wnt^Liver^ cells (Fig. 2E) with a trend towards an increase in M + 3-labeled malate (Fig. 2F).

On the other hand, enrichment of M + 3-labeled citrate, formed when M + 3-labeled OAA combines with unlabeled acetyl CoA (Fig. 2A), was significantly higher in the metM-Wnt^Lung^ as compared to the metM-Wnt^Liver^ cells (Fig. 2G). Notably, the substrate (M + 3 pyruvate) to product (M + 3 citrate or M + 3 OAA) ratio indicated that significantly more M + 3-labeled pyruvate is converted to M + 3-labeled citrate (Fig. 2H) and M + 3-labeled OAA (Fig. 2I), indicating higher PC activity, in the metM-Wnt^Liver^ compared to metM-Wnt^Lung^ cells (*p* < 0.001). In addition, the mRNA level of cytosolic malic enzyme 1 (*Me1*), which converts malate to pyruvate and supports generation of reducing equivalents such as NADPH (Fig. 2A), was significantly higher in metM-Wnt^Liver^ compared to the metM-Wnt^Lung^ cells (*p* = 0.024) (Fig. 2J), potentially increasing production of reducing equivalents from OAA flux. Oxidative stress, induced by H_2_O_2_ treatment, resulted in significantly higher viability of metM-Wnt^Liver^ compared to metM-Wnt^Lung^ cells (*p* = 0.01) (Fig. 2K). Additionally, treatment of exogenous OAA (2 mM) significantly rescued viability of the H_2_O_2_-treated metM-Wnt^Lung^ cells (*p* = 0.0002) (Fig. 2L). These results suggest that the pyruvate-derived OAA in metM-Wnt^Liver^ cells produce malate, while citrate is produced from pyruvate-derived OAA in metM-Wnt^Lung^ cells. Overall, results indicate that metM-Wnt^Liver^ cells have higher PC activity, resulting in enhanced oxidative stress protection via production of mitochondrial OAA.

**Fig. 3:**
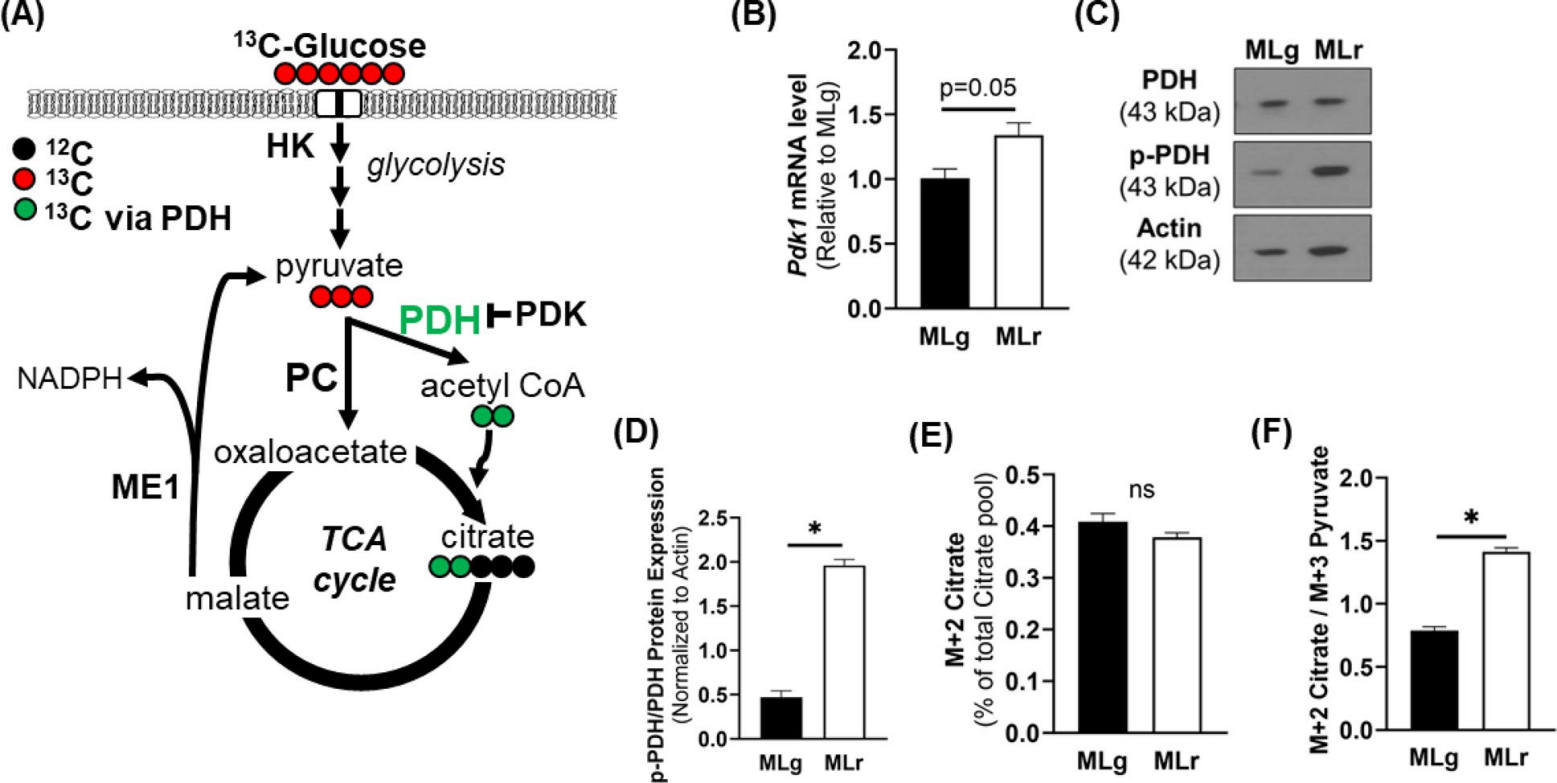
Glucose metabolism via pyruvate dehydrogenase into the tricarboxylic acid cycle. (**A**) Schematic diagram of ^13^C_6_-glucose flux into tricarboxylic acid cycle intermediates. (**B**) mRNA abundance of pyruvate dehydrogenase kinase 1 normalized to 18 S level. (**C**) Representative Western blot and (**D**) quantification of pyruvate dehydrogenase and phospho-pyruvate dehydrogenase protein levels. All band intensities of target protein were normalized to actin. (**E**) ^13^C_6_-glucose incorporation into M + 2-labeled citrate, indicative of pyruvate dehydrogenase activity. (**F**) M + 2 citrate/M + 3 pyruvate indicating pyruvate flux into tricarboxylic acid cycle through pyruvate dehydrogenase. Data are presented as mean ± SEM (*n* = 4). MLg = metM-Wnt^Lung^; MLr = metM-Wnt^Liver^. * *p* < 0.05; ns – not significant

The other major pathway for pyruvate metabolism is through the PDH complex, which catalyzes the irreversible oxidative decarboxylation of pyruvate to form acetyl-CoA and is negatively regulated by PDH kinase (PDK1) phosphorylation (Fig. 3A). *Pdk1* mRNA abundance was 34% higher in metM-Wnt^Liver^ compared to metM-Wnt^Lung^ cells (Fig. 3B). Consistent with these results, protein expression of total PDH is higher in the metM-Wnt^Lung^ cells, whereas phosphorylated PDH is significantly lower in the metM-Wnt^Lung^ cells (Fig. 3C). Overall, there is a decreased phospho-PDH to total PDH ratio in the metM-Wnt^Lung^ cells compared to metM-Wnt^Liver^ cells (*p* = 0.0001) (Fig. 3D). Nonetheless, metabolic tracing of universally labeled ^13^C_6_-glucose indicated that enrichment of M + 2-labeled citrate generated through PDH activity (Fig. 3A) was not different between the metastatic cell lines (Fig. 3E). However, the ratio of M + 2-labeled citrate from M + 3-labeled pyruvate flux into TCA cycle was significantly higher in metM-Wnt^Liver^ cells (Fig. 3F) indicating less glycolysis than metM-Wnt^Lung^ cells. Overall, results showed that PDH activity is similar between the metastatic cell lines despite higher p-PDH/PDH ratio in the metM-Wnt^Liver^ compared to metM-Wnt^Lung^ cells, but higher ratio of pyruvate fluxes into the TCA cycle through citrate in metM-Wnt^Liver^ cells.

### Viability and metabolic adaptations in high glucose concentrations

**Fig. 4:**
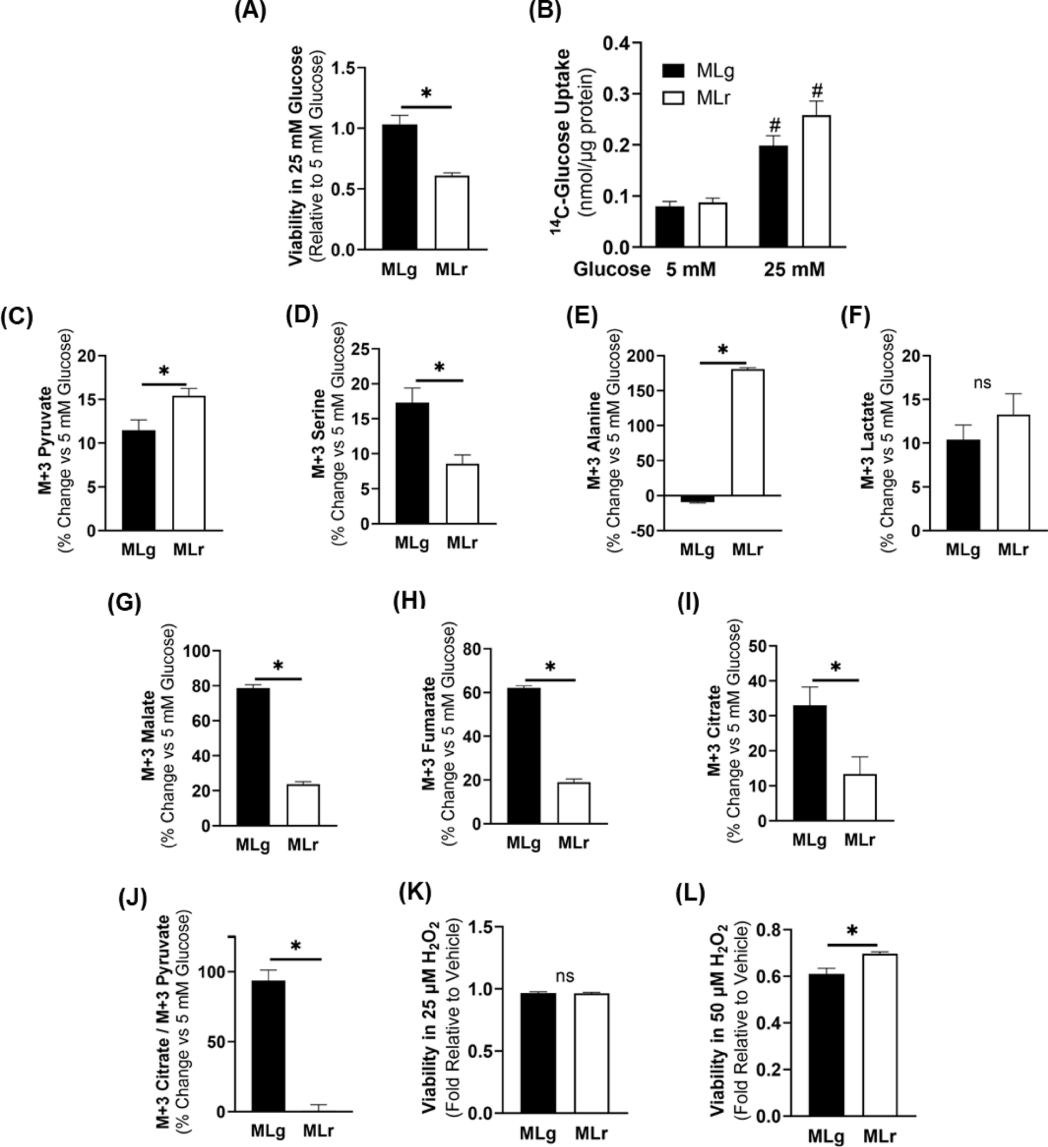
Viability and metabolic adaptations in high glucose concentrations. (**A**) Viability of the cell lines after treatment with high glucose (25 mM) relative to physiologically normal glucose (5 mM) for 48 hrs. (**B**) ^14^C-glucose uptake with 5 and 25 mM glucose, normalized to protein. Percent change in ^13^C_6_-glucose incorporation into (**C**) M+3-labeled pyruvate, (**D**) M+3-labeled serine, (**E**) M+3-labeled alanine, (**F**) M+3-labeled lactate, (**G**) M+3-labeled malate, (H) M+3-labeled fumarate, and (**I**) M+3-labeled citrate upon high glucose treatment. (**J**) Percent change in labeling ratios of M+3 citrate/M+3 pyruvate indicative of pyruvate flux into tricarboxylic acid cycle through pyruvate carboxylase upon high glucose treatment. (**K**) Viability of the cell lines after treatment of 25 µM and (**L**) 50 µM H_2_O_2_ for 24 hrs maintained in 25 mM glucose for total of 48 hrs. Data are presented as mean±SEM (n=4 for radiolabeled uptake and isotope tracing; n=5 for viability assays). MLg = metM-Wnt^Lung^; MLr= metM-Wnt^Liver^*.* * p < 0.05; # p < 0.05 compared to 5 mM glucose; ns – not significant

To investigate whether the observed differences in glucose metabolism affect the ability of the cells to adapt to high glucose levels, cells were treated with 25 mM glucose for 48 h. Results indicate that the metM-Wnt^Lung^ cells remained viable, while metM-Wnt^Liver^ cells had significantly reduced viability when exposed to high glucose (Fig. 4 A). Consistent results were obtained using crystal violet staining to assess cell viability (data not shown). Also, both metastatic cell lines increased glucose uptake at 25 mM glucose concentration at similar levels (Fig. 4B), suggesting that the differential glucose metabolic adaptations under high glucose occur downstream of glucose uptake. Metabolic tracing using 25 mM ^13^C_6_-glucose revealed that metM-Wnt^Lung^ cells significantly reduced M + 3-labeled pyruvate and M + 3-labeled alanine, but significantly increased M + 3-labeled serine under high glucose concentration (Fig. 4C–E). No statistically significant difference was observed for glucose flux to M + 3-labeled lactate (Fig. 4 F). These results suggest that the metM-Wnt^Liver^ cell adaptations to high glucose were more glycolytic compared to the metM-Wnt^Lung^ cells. Interestingly, ^13^C_6_-glucose flux into TCA cycle metabolites malate, fumarate, and citrate with M + 3 labeling pattern were significantly increased in metM-Wnt^Lung^ cells (Figs. 2A and A and 4G–I), suggesting increased TCA cycle flux through PC activity in metM-Wnt^Lung^ cells exposed to high glucose. Consistent with these results, the ratio of M + 3-labeled citrate to M + 3-labeled pyruvate, indicative of PC activity, was significantly higher by 93% in the metM-Wnt^Lung^ than metM-Wnt^Liver^ cells (Fig. 4 J) demonstrating a shift in PC activity in response high glucose conditions between metastatic cell lines. Furthermore, both metastatic cell lines showed similar, minimal effects after exposure to oxidative stress using 25 µM H_2_O_2_ (Fig. 4 K), however, a higher concentration of H_2_O_2_ (50 µM) was required to achieve significant reduction in viability in the metM-Wnt^Lung^ cells (*p* = 0.01) (Fig. 4L). These results suggest that high glucose conditions enhanced the oxidative stress protection in metM-Wnt^Lung^ cells in part by upregulating pathways involved in producing reducing equivalents. Together, these results suggest that the metM-Wnt^Lung^ cells adapt to high glucose concentration in part by increasing glucose flux into the TCA cycle through PC and serine synthesis resulting in improved oxidative stress protection.

### Viability and metabolic adaptations in low glucose concentrations

**Fig. 5:**
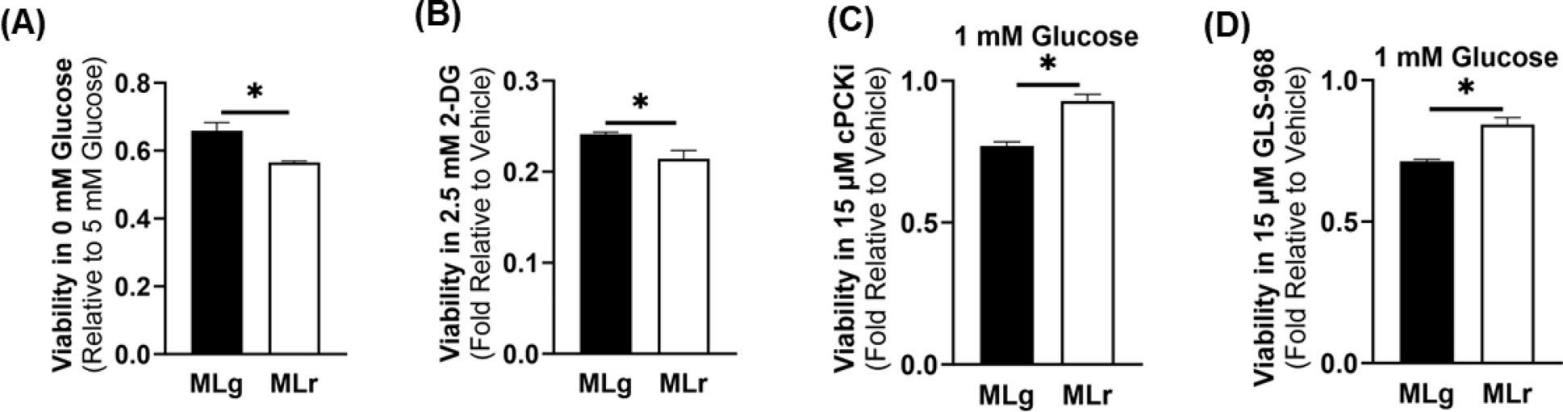
Viability and metabolic adaptations in low glucose concentrations. (**A**) Viability of the cell lines after treatment with glucose-free media relative to physiologically normal glucose, (**B**) treatment with 2.5 mM 2-deoxy-D-glucose relative to vehicle (water) in physiologically normal glucose, (**C**) treatment with 15 µM PCKi in low glucose (1 mM), and (**D**) treatment with 15 µM GLS-968 in low glucose for 48 h. Data are presented as mean ± SEM (*n* = 5). MLg = metM-Wnt^Lung^; MLr = metM-Wnt^Liver^. * *p* < 0.05.

To further characterize the adaptability of the two metastatic cell lines under study herein, differential metabolic adaptation under low glucose conditions was investigated. Results revealed that glucose-free media as well as glucose and 2DG-containing media reduced the cell viability of both metastatic cell lines, although metM-Wnt^Lung^ cells were more viable compared to the metM-Wnt^Liver^ cells (Fig. 5A–B). Further, metM-Wnt^Lung^ cell viability was significantly lower compared to the metM-Wnt^Liver^ cells upon gluconeogenesis inhibition via phosphoenolpyruvate carboxykinase (PCK) chemical inhibitor in the presence of low glucose (*p* = 0.0004) (Fig. 5C), suggesting that the metM-Wnt^Lung^ cells utilize gluconeogenesis to sustain viability during glucose deprivation. In addition, inhibiting the initial enzyme for glutamine catabolism, glutamate synthase (GLS), significantly reduced viability of metM-Wnt^Lung^ cells compared to metM-Wnt^Liver^ cells (*p* = 0.0008) (Fig. 5D), suggesting that the metM-Wnt^Lung^ cells utilize the contribution of glutamine for gluconeogenesis in conditions of low glucose. Simultaneous inhibition of PCK and GLS resulted in an additive effect in the reduction of cell viability without significant difference across cell lines (data not shown), suggesting that glutamine may be used for other metabolic pathways, and that availability of both gluconeogenic and glutamine catabolism pathways are necessary for metM-Wnt^Lung^ cell adaptation under low glucose conditions. Overall, results suggest that metM-Wnt^Lung^ cells are more viable in low glucose conditions in part by utilizing glutamine for gluconeogenesis.

## Discussion

The distinct metabolic characteristics of primary breast cancer cells compared to metastases from different organs suggest the occurrence of metabolic reprogramming during metastasis [16, 19, 21]. Metabolic adaptations of breast cancer cells in the context of organ-specific metastasis exposed to varying glucose concentrations remain largely unexplored, in part due to the lack of appropriate animal models. Here, we elucidated the novel metabolic characteristics and adaptations of lung- and liver-homing mammary tumor-cell lines derived from the same parental cell line under nutrient stress and demonstrated the utilization of alternative pathways that support cell viability.

Our results showed that under physiologically normal glucose (5 mM) conditions, glucose-derived carbons were differentially metabolized between the metastatic cell lines. The metM-Wnt^Lung^ cells exhibit greater glycolytic flux to pyruvate and lactate, whereas the metM-Wnt^Liver^ cells exhibit higher glucose flux into the TCA cycle and PC activity. Notably, these metabolic characteristics shifted between the metastatic cell lines in response to high glucose levels. With high glucose (25 mM) levels, the metM-Wnt^Lung^ cells showed higher glucose flux into the TCA cycle and PC activity while the metM-Wnt^Liver^ cells adapted towards a glycolytic phenotype.

These results are similar to what has been observed in 4T1-derived lung and liver metastatic breast cancer cells grown in DMEM containing high glucose (25 mM), where 4T1-derived liver metastatic breast cancer cells are more glycolytic, while lung metastatic breast cancer cells favor oxidative phosphorylation [16]. Further, the observed upregulation of PC activity in the metM-Wnt^Lung^ cells under high glucose levels is in agreement with studies demonstrating the elevated PC levels in lung metastases and in lung-tropic breast cancer cell models grown in 25 mM glucose [29–31]. For instance, the lung microenvironment is enriched in pyruvate [20], and 4T1 lung metastases exhibit elevated PC-dependent anaplerosis, with a dose-dependent increase in PC activity in response to pyruvate treatment [30]. Consequently, inhibiting PC prevents breast cancer pulmonary metastasis in vivo [29, 31]. Our results showed that PC activity is one of the metabolic adaptations utilized by the metM-Wnt^Lung^ cells when exposed to high glucose. It is possible that the elevated PC activity allows the rapid anaplerotic flux to induce PC-mediated pyruvate cycling [32, 33]. Pyruvate cycling, occurring via pyruvate/malate cycle, pyruvate/(iso)citrate cycle, and isocitrate/α-ketoglutarate shuttle, produces NADPH as an end-product instead of NADH in conditions of high glucose [32, 33]. In agreement with this, the metM-Wnt^Lung^ cells displayed improved oxidative stress protection under high glucose conditions. The observed propensity of the metM-Wnt^Lung^ cells to adapt to high glucose levels and elevated pyruvate formation through PC anaplerosis is likely favorable in the pyruvate-enriched and highly oxygenated lung microenvironment. In contrast, the glycolytic adaptation of the metM-Wnt^Liver^ cells under high glucose levels may not be supported in the context of the pyruvate-rich and highly oxygenated lung microenvironment, resulting in the inability of those cells to survive in the lungs. Also, previous literature highlights the presence of hypoxic regions in the liver microenvironment [7, 16]. Thus, it is possible that the shift towards a glycolytic phenotype in the metM-Wnt^Liver^ cells is a metabolic adaptation conducive for successful colonization of the hepatic instead of the pulmonary microenvironment. Although the use of supraphysiologic glucose (25 mM) in cell culture is widely used in vitro, such glucose levels may not replicate the elevated glucose level encountered by cancer cells in vivo, but none-the-less demonstrate the adaptability to higher glucose concentrations, and may also vary depending on the actual glucose availability.

Glucose deprivation is common among neoplastic tumor cells due to its high rate of glucose demand and nutrient deprivation conditions as the tumor grows [34]. Although gluconeogenesis was previously considered to be limited to gluconeogenic organs, several studies have demonstrated the occurrence of gluconeogenesis in diverse tumors, likely to maintain the levels of precursors for biomass production under glucose deprivation [34]. With glucose and glutamine being two of the major energy sources, cancer cells often compensate for starvation from either of these nutrients by increasing utilization of the other nutrient to sustain metabolic activities [35]. Our work demonstrated that the metM-Wnt^Lung^ cells are more adaptable to maintain cell viability through gluconeogenesis and glutamine catabolism while the metM-Wnt^Liver^ cells are more glucose-dependent during glucose deprivation. Indeed, it is likely that glutamine is utilized for the gluconeogenic pathway to fuel biosynthetic pathways that are normally sustained by glucose such as nucleotide synthesis via serine *de novo* formation and one-carbon metabolism during glucose deprivation, as reported [34, 36]. Previous work also reported similar utilization of glutamine in human B cell model of Burkitt lymphoma (P493) which provided anabolic precursors such as citrate for lipid synthesis and cell survival precursors for glutathione production during glucose deprivation [37]. Conversely, glycolysis inhibitors, such as 2-DG, have been tested as an adjunct therapy in cancers such as breast cancer [38]. However, glycolysis inhibitors have generally shown limited success in clinical trials due to their non-selectivity and the ability of cancer cells to reprogram metabolism by activating alternative pathways, including glutamine metabolism or detoxifying 2-DG through enzymes such as Dog2, leading to reduced therapeutic efficacy and potential tumor recurrence [38]. Our study provides insights into these adaptive mechanisms by revealing the contribution of alternative pathways, including gluconeogenesis and glutamine metabolism, to support TNBC survival when glucose is limited.

Our results underscore the importance of targeting metabolic plasticity in TNBC to prevent metastasis at multiple sites. The distinct adaptations observed in lung- and liver-tropic cells suggest that glycolysis inhibition alone is unlikely to be effective as cancer cells, specifically lung-tropic breast cancer cells, can compensate through pathways such as gluconeogenesis and glutamine metabolism to maintain energy homeostasis and support survival. We also found that excess glucose, which can occur in hyperglycemic states or supraphysiologic culture conditions, differentially affects metastatic cell lines, where lung-tropic cells are more adaptable. Together, these findings indicate that both glucose deprivation and excess influence metabolic adaptation which are vulnerabilities where multiple targets may need to be considered to be most effective in preventing metastasis.

## Conclusion

**Fig. 6:**
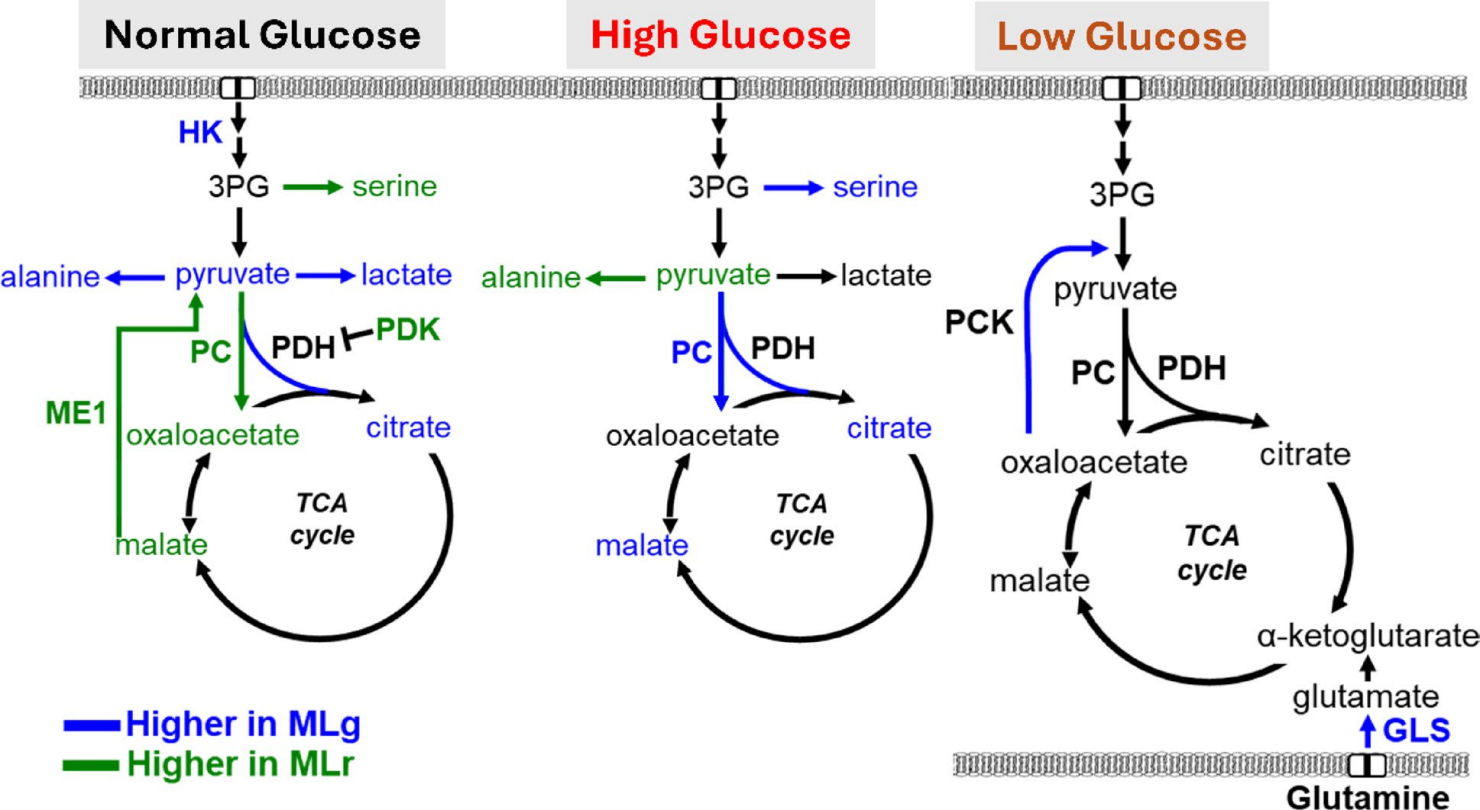
Metabolism and adaptability in varying glucose levels. HK: hexokinase; 3PG: 3-phosphoglycerate; PC: pyruvate carboxylase; PDH: pyruvate dehydrogenase; PDK: pyruvate dehydrogenase kinase; ME1: cytosolic malic enzyme 1: PCK: pyruvate carboxykinase; GLS: glutamate synthase

Taken together, our results demonstrate the unique metabolic characteristics and adaptive mechanisms of lung- versus liver-homing TNBC cell lines in response to varying glucose levels (Fig. 6). Specifically, metM-Wnt^Lung^ cells, unlike metM-Wnt^Liver^ cells, can adapt to varying glucose concentrations by shifting metabolism from glycolysis to oxidative phosphorylation under glucose excess, and by utilizing glutamine and gluconeogenesis under glucose deprivation. Given the current lack of targeted therapy options for metastatic TNBC, our preclinical findings of metabolic vulnerabilities underlying metastatic potential that can be exploited as therapeutic targets represent an important first step towards developing novel treatment strategies for reducing the burden of metastasis in women with TNBC.

## Data Availability

No datasets were generated or analysed during the current study.

## Abbreviations

TCA: tricarboxylic acid
2DG: 2-deoxy-D-glucose
DMSO: Dimethyl sulfoxide
PCKi: Phosphoenolpyruvate carboxykinase inhibitor
PDH: Pyruvate dehydrogenase
DMEM: Dulbecco’s Modified Eagle Medium
FBS: Fetal bovine serum
PBS: Phosphate-buffered solution
HK: Hexokinase
G3P: Glyceraldehyde-3-phosphate
3PG: 3-phosphoglycerate
PC: Pyruvate carboxylase
OAA: Oxaloacetate
ME1: Cytosolic malic enzyme 1
PDK1: Pyruvate dehydrogenase kinase 1
PCK: Phosphoenolpyruvate carboxykinase
GLS: Glutamate synthase
